# A visual security multi-key selection image encryption algorithm based on a new four-dimensional chaos and compressed sensing

**DOI:** 10.1038/s41598-024-66320-6

**Published:** 2024-07-05

**Authors:** Shuqin Zhu, Congxu Zhu

**Affiliations:** 1 School of Computer Science and Technology, Liaocheng University, Liaocheng, 252059 China; 2 School of Computer Science and Engineering, Central South University, Changsha, 410083 China

**Keywords:** Engineering, Mathematics and computing

## Abstract

In this article, a visual security image encryption algorithm based on compressed sensing is proposed. The algorithm consists of two stages: the compression and encryption stage and the embedding stage. The key streams in the compression and encryption stage are generated by a newly constructed four-dimensional discrete chaotic map, while the Gaussian measurement matrix is generated by a Chebyshev map, and both of their generations are related to the feature code of the carrier image, which enhances the security of the ciphertext. In the compression and encryption stage, a scrambling-cyclic shift-diffusion encryption structure is adopted for the compressed image in which the shift number in the cyclic shift stage and the diffusion key streams are dynamically changed according to each pixel value, so the algorithm can resist chosen plaintext attack. In the embedding stage, the carrier image is first subjected to integer wavelet transform to obtain the high-frequency and low-frequency components of the image, and then the intermediate ciphertext information is embedded into its high-frequency components. Finally, the carrier image is subjected to inverse integer wavelet transform to obtain a visually secure ciphertext image. The experimental results and security analysis indicate that the encryption scheme has a large key space, high decryption key sensitivity, similar histogram distribution between the carrier image and the visual security ciphertext image, and good robustness to noise attacks.

Image information security is the process of protecting digital image content from unauthorized access, modification, or leakage. With the popularization of digital media and the development of Internet technology, image information security has become particularly important. For example, many images contain personal or sensitive information, such as identity photos, family photos, etc. If these images are accessed by unauthorized individuals, it may lead to privacy breaches. Artists, photographers, and other creative workers rely on their visual works to earn income. If these images are illegally copied or distributed, it will harm their economic interests and creative rights. In fields such as law, medical diagnosis, and scientific research, the authenticity and completeness of images are crucial. Any unauthorized modifications may lead to incorrect judgments or decisions. Furthermore, malicious software (such as viruses and Trojans) can spread through image files, and once a user opens an infected image, it may pose a threat to the device or system. Tampering images may mislead the public, affecting public opinion and social trust. Therefore, image information security is crucial for protecting personal privacy, maintaining copyright, ensuring data integrity, and preventing security risks.

The most effective way to protect image information security is to encrypt images. But currently, almost all image encryption schemes encrypt plaintext images into ciphertext images similar to noise or texture. This type of ciphertext image can easily attract the attention of hackers during transmission or storage, and then be targeted for attack. Therefore, how to design a visual secure image encryption algorithm, that is, the obtained ciphertext image is not disorderly but visually meaningful, and can ensure both data and visual security of information, has become a research hotspot with high research value. Bao and Zhou^1^ proposed a visually secure image encryption method for the first time, in which a plaintext image was encrypted into a noisy ciphertext image, and then the carrier image was subjected to discrete wavelet transform (DWT) to obtain *LL*, *LH*, *HL* and *HH* components and replace the *LH* and *HL* components of the carrier image with the integer and remainder parts of the ciphertext image. In reference^2,3^, the plaintext image was pre encrypted into a ciphertext image, and then the ciphertext image was embedded into the carrier image to obtain a visually secure ciphertext image. Although these algorithms can achieve visual security, the size of ciphertext is too large, which is four times the size of plaintext images, seriously increasing the burden of storage space and transmission bandwidth. If the encrypted image can be further compressed before transmission, it can not only improve transmission efficiency, but also be more suitable for bandwidth limited transmission channels. Fortunately, the emergence of compressive sensing (CS) has made it possible to perform both compression and encryption simultaneously. Since the proposal of compressive sensing in 2006, many scholars have combined chaotic systems with compressive sensing to design many image encryption algorithms^4–10^, as well as some visual security encryption algorithms^11–14^. For example, in reference^11^, Chai et al. designed a single grayscale visual security image encryption algorithm using CS and chaotic systems, ensuring that the ciphertext image is the same size as the plaintext image, and the algorithm is related to the hash value of the plaintext image, effectively resisting chosen (known) plaintext attacks. In reference^13^, an efficient and robust meaningful image encryption (MIE) scheme was proposed by combining block compression sensing (BCS) and singular value decomposition (SVD) embedding. Yuan di Shi et al. proposed a visual security image encryption scheme based on adaptive block compression sensing and non negative matrix decomposition^14^, in which experimental results show that this scheme can balance the security of image information transmission and the integrity of received ciphertext and has good resistance to various attacks. Zhou et al.^15^ proposed an efficient image compression and encryption scheme based on hyperchaotic systems and two-dimensional compression sensing, in which , the plaintext image is compressed and encrypted simultaneously using measurement matrices in both directions. Then, the obtained ciphertext image was subjected to a cyclic shift operation method controlled by the hyperchaotic system, which can effectively change pixel values. These proposed encryption algorithms reduced data transmission volume, greatly improved the transmission efficiency.

In references^1–3,11^, ciphertexts were directly embedded into the carrier image, which directly leads to a too concentrated pixel value distribution in the ciphertext image and poor security. For a good encryption algorithm, we prefer to obtain a uniform pixel value distribution in the ciphertext image, which can increase the security of the algorithm. Due to the low correlation between algorithms and plaintext, many encryption algorithms have been cracked^16–21^. Therefore, effective information from plaintext images should be utilized to design image encryption algorithms to improve the security of the algorithms. At present, most image encryption algorithms adopt information related to plaintext images, such as pixel average^22^, hash value^11^, information entropy^23^, to design keys to increase the connection between the algorithm and plaintext and improve the algorithm's resistance to chosen plaintext attacks. However, during decryption, it is necessary to transmit these parameters related to plaintext information to the receiver. When encrypting a large number of images at the same time, an additional amount of information needs to be transmitted, which will cause serious transmission burden. In addition, once these parameters are stolen by hackers during the transmission process, they are likely to crack plaintext images, so these algorithms have certain security risks.

Both high-frequency and low-frequency components can be obtained by performing lifting wavelet transform (LWT)^2^ or discrete wavelet transform (DWT)^1,3,11^ on the carrier image. However, LWT and DWT transformations are not completely reversible, which leads to energy loss when performing inverse operations on carrier images embedded with the intermediate ciphertext information, so the extracted information during decryption differs from the embedded data, resulting in poor quality of reconstructed images. Therefore, it is urgent to find a better wavelet transform that can achieve complete reversibility without energy loss when performing inverse operations on carrier images.

Therefore, in order to solve the above problems, this article proposes a new visual secure image encryption algorithm, which has the following advantages:

1. Design a method for extracting carrier image feature codes, which are robust and unique.
2. The initial values of the chaotic system are set to be related to the feature code of the carrier image, so the key streams generated by the chaotic system are related to the carrier image. When decrypting, due to the robustness of the feature code, the receiver only needs to directly extract the feature code of the received ciphertext image, without the need to transmit the feature code of the carrier image separately.
3. In the encryption phase, an image encryption algorithm with dynamic key selection mechanism was adopted. According to the different pixel positions, different random sequences are selected for encryption to further ensure the algorithm's resistance to plaintext/ciphertext attacks
4. In the embedding stage, integer wavelet transform (IWT) is applied to the carrier image to solve the problem of energy loss during the reconstruction of discrete wavelet transform and lifting wavelet transform.

In addition, the highlights of this paper also include the following two points:

1. A four-dimensional discrete chaotic map was constructed based on Marotto's theorem and it was theoretically proved that the map is chaotic in in the Li Yorke sense.
2. A method for constructing a Gaussian measurement matrix based on chaotic mapping is proposed, which can quickly and massively generate a Gaussian measurement matrix. The generated Gaussian measurement matrix can be conveniently applied in compressed sensing.

## Preliminary knowledge

The basic technologies used in encryption systems are introduced to lay the groundwork for designing encryption algorithms.

### Visual security encryption technology

Visual security encryption technology is also known as Visual Cryptography. It is an information hiding technique that allows secret information to be visually hidden in images, so that only people with specific keys can interpret the hidden information, while others cannot detect the existence of the information. The usefulness of visual cryptography lies in its provision of a simple and effective method to protect information from unauthorized access. Compared with traditional encryption techniques, visual cryptography does not require complex algorithms or computing power, but relies on patterns and color changes that are difficult for the human eye to recognize to achieve information hiding. Here are some examples of visual cryptography and their application scenarios.

1. Secret image transmission: Important images or files can be converted into seemingly ordinary images and sent via email or social media without attracting the attention of third parties. It can be used for the covert transmission of sensitive information, such as military communication, trade secret protection, etc.
2. Data privacy protection: Companies or individuals can use visual cryptography technology to protect their private images or data, such as hiding identity documents, credit card information, etc. in seemingly harmless images. It can prevent personal information leakage and increase data security.
3. Digital watermarking: Embedding invisible marks in digital media (such as images, videos, audio), which can be copyright information, owner identity, etc. This can be used for copyright protection and piracy tracking to ensure the security of intellectual property.
4. Security certification: Print specific visual patterns on documents or product packaging that may appear random to the naked eye, but can be verified for authenticity through specific decoding methods. This can be used for anti-counterfeiting identification, such as anti-counterfeiting labels for banknotes, passports, branded goods, etc.
5. Access Control: Use visual passwords to generate a card or label containing secret information, and only personnel who know the decoding method can obtain access permissions. This can be used for physical and logical access control systems, such as access control systems, computer login, etc.

### The construction of a new four dimensional discrete chaotic map

In this chapter, a new four-dimensional discrete chaotic map is constructed based on the Marotto theorem, and a pseudo-random number generator is designed based on this four-dimensional chaotic map. The random sequences used in the plaintext image encryption algorithm in the following chapters are all generated by the random number generator.

Theorem 1 Marotto's theorem^24^.

Let *z* ∈ *R*^*n*^ be a fixed point for the mapping *f* :*R*^*n*^ → *R*^*n*^, assuming:

1. *f* is continuously differentiable in a certain field of *z* and the absolute values of all eigenvalues of *Df*(*z*) are greater than 1, thus there exists a normal number* r.*
  And a norm ‖·‖of *R*^*n*^ , such that* f* expands on *B*_*r*_ (*z*) below the norm‖·‖ of *R*^*n*^, where *B*_*r*_ (*z*) is a closed sphere centered on z in space (*R*^*n*^*,*‖·‖).
2. *z* is a return extension fixed point of *f*, that is, there exists a point *x*_0_ ∈ *B*_*r*_(*z*) and a positive integer *m*, satisfying *f*^*m*^(*x*_0_) = z, where *B*_*r*_ (*z*) is a closed sphere centered on z in space (*R*^*n*^*,*‖·‖), *f* is continuously differentiable in a certain fields of *x*_0_,*x*_1_,…*x*_*m*–1_and satisfies det*Df*(*x*_*j*_) ≠ 0, where *x*_*j*=_*f*(*x*_*j*–1_), (0 ≤ *j* ≤ *m*–1). Then the mapping *f* is chaotic in the Li Yorke sense.

The form of the four dimensional discrete chaotic system constructed based on Theorem 1 is shown in Eq. (1)

1 $$\left\{ {\begin{array}{*{20}l} {x_{1} \left( i \right) = {\text{sin}}\left( {x_{1} \left( {i - {1}} \right)} \right) \times {\text{sin}}\left( {x_{2} \left( {i - {1}} \right)} \right) - a \times \sin \left( {x_{4} \left( {i - {1}} \right)} \right);} \hfill \\ {x_{2} \left( i \right) = b \times {\text{sin}}\left( {x_{1} \left( {i - {1}} \right)} \right) \times {\text{cos}}\left( {x_{2} \left( {i - {1}} \right)} \right) - x_{1} \left( {i - {1}} \right)} \hfill \\ {x_{3} \left( i \right) = c \times x_{2} \left( {i - {1}} \right) + t \times {\text{sin}}\left( {x_{3} \left( {i - {1}} \right)} \right);} \hfill \\ {x_{4} \left( i \right) = d \times {\text{sin}}\left( {x_{2} \left( {i - {1}} \right)} \right) + \sin x_{4} \left( {\left( {i - {1}} \right)} \right);} \hfill \\ \end{array} } \right.$$

Among them, the parameters are *a* = *b* = 4, *c* = 3.5, *d* = 2, *t* = 4. The sequences generated by this system are chaotic sequences. The following proof shows that the mapping satisfies the Marotto theorem.

#### Proof

Obviously,* z* = (0,0,0,0) is a fixed point of the mapping (1), and for the mapping (1) with *r* > 0, it is continuously differentiable on *B*_*r*_(*z*). The expression of its Jacobian matrix is *Df*(*x*).

*Df* (*x*) = $$\left[ {\begin{array}{*{20}l} {\cos (x_{1} (i - 1))\sin (x_{2} (i - 1))} & {\cos (x_{2} (i - 1))\sin (x_{1} (i - 1))} & 0 & { - a\cos (x_{4} (i - 1))} \\ {b{\text{cos}}(x_{1} (i - 1))\cos (x_{2} (i - 1))} & { - b{\text{sin}}(x_{1} (i - 1))\sin (x_{2} (i - 1))} & 0 & 0 \\ 0 & c & {t\cos (x_{3} (i - 1))} & 0 \\ 0 & {d\cos (x_{2} (i - 1))} & 0 & {\cos (x_{4} (i - 1))} \\ \end{array} } \right]$$

Then take *z* = (0,0,0,0) into *Df* (*x*), and obtain.

*Df*(z) = $$\left[ {\begin{array}{*{20}l} 0 & 0 & 0 & { - {\text{a}}} \\ {b - 1} & 0 & 0 & 0 \\ 0 & c & t & 0 \\ 0 & d & 0 & 1 \\ \end{array} } \right]$$

The four eigenvalues of the matrix *Df*(z) obtained by calculation are 4, -2.5868, 1.7934 + 2.4621*i*, and 1.7934–2.4621*i*, respectively, indicating that the absolute values of all eigenvalues are greater than 1.Therefore, there exists a normal number* r* and a norm ‖·‖of *R*^*n*^ , such that* f* expands on *B*_*r*_ (*z*) below the norm‖·‖ of *R*^*n*^,

According to the calculation, it is found that there exists *x*_0_ = (0,0,arcsin($\pi$/4),0) ≠ *z* and a positive integer *m* = 2, so that *f*^2^ (*x*_0_) = *z* = 0. Where *x*_1_ = *f*(*x*_0_) = (0,0,$\pi$,0), and

det(D*f*(*x*_0_)) = $$\left| {\begin{array}{*{20}l} 0 & 0 & 0 & { - {\text{a}}} \\ {b - 1} & 0 & 0 & 0 \\ 0 & c & {t\cos (\arcsin (\pi /4))} & 0 \\ 0 & d & 0 & 1 \\ \end{array} } \right|$$ = − 59.4231

det(D*f*(*x*_1_)) = $$\left| {\begin{array}{*{20}l} 0 & 0 & 0 & { - {\text{a}}} \\ {b - 1} & 0 & 0 & 0 \\ 0 & c & { - t} & 0 \\ 0 & d & 0 & 1 \\ \end{array} } \right|$$ = 96

So* f* is continuously differentiable in a certain fields of *x*_0_, *x*_1_and satisfies det*Df*(*x*_*j*_) ≠ 0, where *x*_*j*_ = *f*(*x*_*j*–1_), (0 ≤ *j* ≤ *m*–1). Then the mapping *f* is chaotic in the Li Yorke sense.

**Figure 1:**
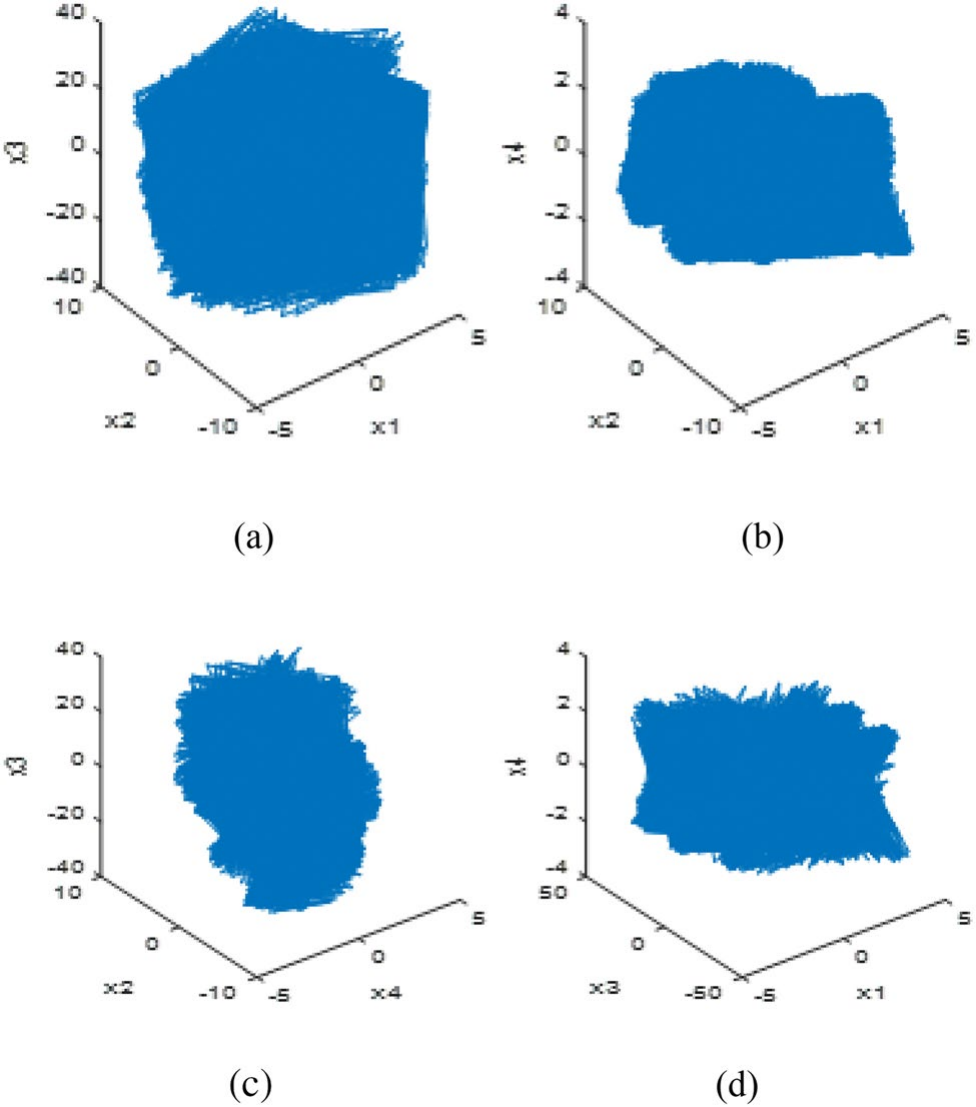
The running track of chaotic system (1). (**a**) *x*_1_(*k*)−*x*_2_(*k*)−*x*_3_(*k*), (**b**) *x*_1_(*k*)−*x*_2_(*k*)−*x*_4_(*k*), (**c**) *x*_4_(*k*)−*x*_2_(*k*)−*x*_3_(*k*), (**d**) *x*_1_(*k*)−*x*_3_(*k*)−*x*_4_(*k*).

The maximum Lyapunov exponent of chaotic map (1) calculated using the Wolf method is 0.8976, which also verifies that the map is a chaotic mapping. When the initial value is given as *X*(0) = {1.7325, 0.8367, 1.5872, 0.8632}, Chaotic orbits of the variables of system (1) are shown in Fig. 1.

### Design and performance analysis of a pseudorandom number generator based on the chaotic mapping(1)

Firstly, construct a pseudo-random number generator based on this chaotic mapping. Set the initial values *x*_1_(0), *x*_2_(0), *x*_3_(0), *x*_4_(0) of the chaotic system (1) to generate four chaotic sequences of length *l*, ***x***_***t***_ = {*x*_*t*_(*k*)|*k* = 1, 2, 3 *,…,l*;* t* = 1, 2, 3, 4} and the sequences *x*_*i*_, *x*_*j*_, *x*_*q*_ are used to generate key streams ***T***_***i,j,q***_ of length *l* according to Eq. (2). Here, *i*, *j, q* are not equal to each other, and *i*,* j*, *q* {1, 2, 3, 4}, thus obtaining *C*_*4*_^3^ = 4 different sequences *T*_*i. j, q.*_ (*i, j, q* in ascending order).

2 $$T_{i,j,q} (X(k)) = \bmod \left( {round\left( {\frac{255\sqrt 2 L(X(k) - \min (X))}{{\max (X) - \min (X)}}} \right),256} \right)$$

where $X(k) = k_{1} x_{i} (k) + k_{2} x_{j} \left( k \right)x_{q} (k)$, $k_{1} = \sqrt 3$, $k_{2} = \sqrt 5$, $L = 10^{10}$, $\min (X) = \min \{ X(k)|k = 1,2,...,l\}$, $\max (X) = \max \{ X(k)|k = 1,2,...,l\}$, Round(*x*) represents taking the integer closest to *x*. The initial value of a chaotic system serves as the seed of a random number generator, so different initial values of the chaotic mapping (1) result in four different random number sequences *T*_*i, j, q*_. These four random sequences are combined into six pairs of random sequences, as shown in Table 1. Different random sequence pairs from Table 1 are chosen when encrypting pixels at different positions. This is the principle of the key dynamic selection mechanism.

**Table 1:** Key stream table.

Key pair	0	1	2	3	4	5
*Key*_1_	*T*_1,2,3_	*T*_1_*,*_2,3_	*T*_1,2,3_	*T*_1,2,4_	*T*_1,2,4_	*T*_1,3,4_
*Key*_2_	*T*_1,2,4_	*T*_2,3,4_	*T*_1,3,4_	*T*_2,3,4,_	*T*_1,3,4_	*T*_2,3,4_

1. Pseudorandomness detection of SP800-22

The NIST (National Institute of Standards and Technology, USA) SP800-22 is a standard test software package to evaluate the randomness performance of time series^25^. These tests focus on various types of non randomness that may exist in the sequence, some of which can be decomposed into various seed tests. 16 test items include frequency test, block frequency test, run test, binary matrix rank test, etc. Before the test, a significance level *α* is set. It requires multiple groups of sequence (at least 100 groups) to be tested, and the length of each group is 1,000,000 bits. Therefore, the total length of 100 groups of sequences is 100 × 1,000,000 bits. There are two performance indicators, the p-value and pass rate, that are employed to measure the stochastic performance of time series. The default significant level *a* = 0.01. The confidence interval that is used to test the pass rate is defined as 1 − *α* − 3 × [*α*(1 − *α*)/*m*]^1/2^ to 1 − *α* + 3 × [*α*(1 − *α*)/*m*]^1/2^, where *m* is the number of groups of bit sequences. When *α* = 0.01 and *m* = 100, the confidence interval is [0.9602, 1.0198], which indicates that the minimum passing rate must be 96%.

To perform randomness detection on the random numbers generated by the pseudo-random number generator. Set the initial values *x*_1_(0) = 1.7325, *x*_2_(0) = 0.8367, *x*_3_(0) = 1.5872, *x*_4_(0) = 0.8632 to obtain four random sequences *T*_1,2,3_, *T*_1,2,4_, *T*_1,3,4_ and *T*_2,3,4_, each sequence has a length of *S*. In order to test the randomness of the sequence with NIST software, four sequences *T*_1,2,3_, *T*_1,2,4_, *T*_1,3,4_ and *T*_2,3,4_ are connected to obtain a sequence **T** with a length of 4*S*, and each element in the sequence is an 8-bit non negative integer. Therefore, the sequence **T** contains a total of 32*S* bits. The significance level is set to *a* = 0.01 and *m* = 100. Let *S* = 3,125,000, then **T** contains a total of 100 × 1,000,000 bits. The 16 indicators test results of NISTSP800-22 are shown in Table 2, in which the respective *P* value and pass rate are listed. It is worth noting that the item marked with * contains multiple sub items. We listed the result with the lowest p-value corresponding to the lowest pass rate. The P-values in the test results are all greater than *a* = 0.01 and the minimum passing rate is above 96%, indicating that the randomness of the chaotic sequence meets requirements of SP800-22.

**Table 2:** NISTSP800-22 Standard Test for random sequence generated by the chaotic map.

The statistical test item	*P* value	Pass rate	Results
Frequency	0.534146	98/100	Pass
Intra block frequency	0.419021	100/100	Pass
Cumulative sums-forward	0.983453	98/100	Pass
Cumulative sums-reverse	0.678686	98/100	Pass
Runs	0.224821	99/100	Pass
Long runs of ones	0.719747	99/100	Pass
Binary matrix rank	0.171867	99/100	Pass
Spectral DFT	0.595549	97/100	Pass
Non-overlapping template*	0.202268	96/100	Pass
Overlapping template	0.867692	100/100	Pass
Universal	0.419021	100/100	Pass
Approximate entropy	0.514124	97/100	Pass
Random excursions*	0.264458	61/63	Pass
Random excursions Variant*	0.116519	62/63	Pass
Serial test 1	0.019188	97/100	Pass
Serial test 2	0.350485	98/100	Pass
Linear complexity	0.534146	98/100	Pass

(2) Information entropy and histogram analysis

**Figure 2:**
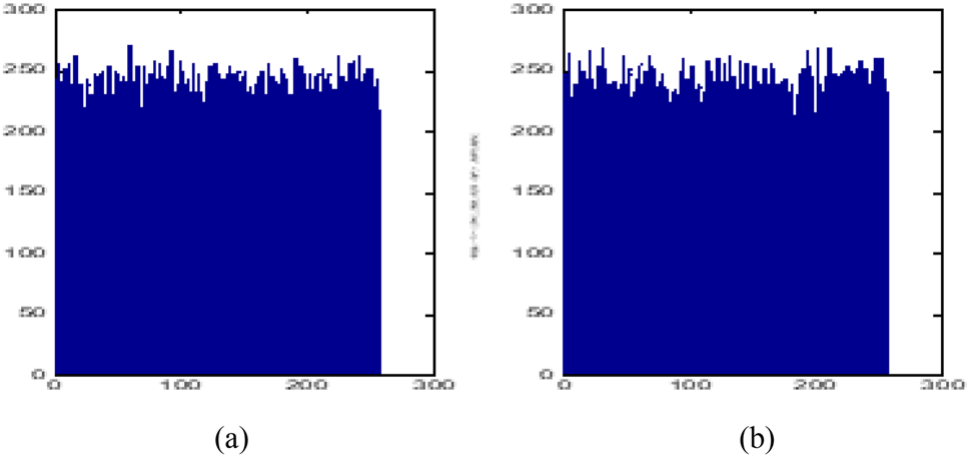
Homogeneity detection of random sequence. (**a**) The distribution of random sequence *T*_1,2,3_; (**b**) The distribution of random sequence *S*_1,2,3_.

Entropy of a message is used to measure the randomness of the message, which is defined by

3 $$E\left( m \right) = - \sum\limits_{i = 1}^{n - 1} {p\left( {m_{i} } \right)} \log_{2} \left( {p\left( {m_{i} } \right)} \right)$$

where *p*(*m*_*i*_) denotes the probability of gray level *m*_*i*._ When the sequence {*m*_*i*_} is of equal probability distribution, it has a maximum entropy of 8 bits. The greater the information entropy of a sequence {*m*_*i*_}, the better its randomness. The calculation result shows that the maximum, minimum, and average values of information entropy in the four sequences *T*_*i,j,q*_ are 7.9978 bit, 7.9477 bit, and 7.99965 bit, respectively, while the maximum, minimum, and average values of information entropy in the four sequences *S*_*i,j,q*_ are 7.9956 bit, 7.9377 bit, and 7.9935 bit, respectively, all very close to the ideal value. The numerical distribution curves of *T*_1,2,3_, S_1,2,3_,are shown in Fig. 2a,b, respectively .It can be seen that the random sequences *T*_1,2,3_ and *S*_1,2,3_ are evenly distributed and have good pseudo randomness.

### The generation of feature code for the carrier image based on singular value decomposition (SVD)

In some encryption systems, algorithms are not sensitive to plaintext images and cannot effectively resist chosen-plaintext attacks. In some algorithms, the hash value or pixel sum of plaintext images are used as a part of the initial value of the chaotic system, which enhances the security of the encryption system. However, when decrypting, both the initial value of the chaotic system and the hash value of the image are required, which increases the difficulty of key management. So it is not feasible in practical applications. To address this issue, this paper proposes a method of extracting feature code based on singular value decomposition (SVD) for carrier images. The feature codes must meet the following conditions

1. Robustness: The feature codes of the original carrier image, the image embedded in the intermediate ciphertext, and the attacked ciphertext image must be the same, that is, the extracted feature codes must be robust to certain common attacks
2. Uniqueness: The feature codes should be different for different images. That is to say, a feature code should uniquely correspond to a specific image.

**Figure 3:**
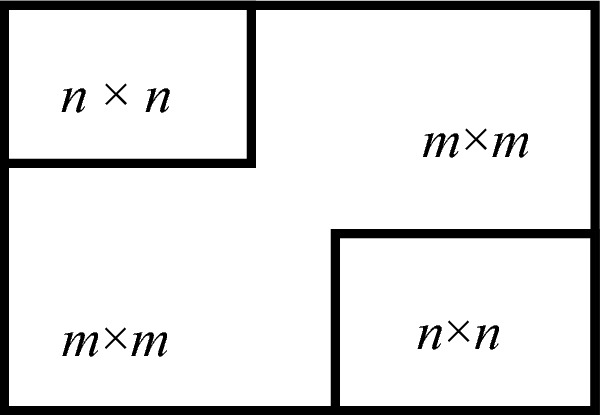
Block diagram: A block with a size of (*m* + *n*)$\times$(*m* + *n*) is divided into two sub blocks.

The steps for extracting feature code are as follows:

1. Assuming the carrier image is I of the size *M* × *N* , divide I into blocks with the size (*m* + *n*) × (*m* + *n*), so the number of blocks is *L* = *M*/(*m* + *n*) × *N*/(*m* + *n*), *m* ≥ *n*, Number these small block images in order of *I*_1,_ *I*_2_, …, *I*_*L*_ from left to right, and from top to bottom. For each block* I*_*k*_ (*k* = 1, 2, … *L*), take its sub block *I*_*k*1_ with size *n* × *n* in the upper left corner and sub block* I*_*k*2_ with size *n* $\times$ *n* in the lower right corner, as shown in Fig. 3.

(2) SVD decomposition for*I*_*k*1_ and *I*_*k*2_, respectively as shown in Eqs. (4) and (5)
  4 $$I_{k1} = U_{k1} S_{k1} V_{k1}^{{\text{T}}}$$
  5 $$I_{k2} = U_{k2} S_{k2} V_{K2}^{T} \left( {k = {1},{2},{3}, \ldots ,L} \right)$$
  where $$U_{ki} \in R^{n \times n} ,$$$$V_{ki} \in R^{n \times n}$$ are all unitary matrices, and $$U_{ki} U_{ki}^{T} = I$$, $$V_{ki} V_{ki}^{T} = I$$. The superscript T represents a matrix transpose, $${\text{S}}_{ki} \in R^{n \times n}$$ is a diagonal matrix. If the rank of the matrix is *r*, then the diagonal elements of $$S_{ki}$$(*i* = 1,2) satisfies $$\sigma_{1} \ge \sigma_{2} \ge .... \ge \sigma_{r} \ge 0$$.
(3) Obtain the maximum values of $S_{k1} ,S_{k2}$ respectively, and record them as $\sigma_{k1} ,\sigma_{k2}$.The maximum feature values obtained from all blocks form a vector $$S = \left( {\begin{array}{*{20}l} {\sigma_{11} } & {\sigma_{12} } & {\sigma_{21} } & {\sigma_{22} } & {\sigma_{31} } & {\sigma_{32} } & {...} & {\sigma_{L1} } & {\sigma_{L2} } \\ \end{array} } \right)$$ in order. and then number the elements in S from left to right to obtain S: $$S = \left( {\begin{array}{*{20}l} {\sigma_{11} } & {\sigma_{12} } & {\sigma_{21} } & {\sigma_{22} } & {\sigma_{31} } & {\sigma_{32} } & {...} & {\sigma_{L1} } & {\sigma_{L2} } \\ \end{array} } \right) = \left( {\begin{array}{*{20}l} {s_{1} } & {s_{2} } & {s_{3} } & {s_{4} } & {s_{5} } & {s_{6} } & {...} & {s_{2L - 1} } & {s_{2L} } \\ \end{array} } \right)$$.
(4) Compare the sizes of any two elements in S to obtain the feature code sequence FC according to the following rules
  6 $$FC_{k} = \left\{ {\begin{array}{*{20}l} {1,s_{i} > s_{j} } \\ {0,s_{i} \le s_{j} } \\ \end{array} } \right.i = 1,2,3,...2L - 1,j > i$$
  Therefore, the length of FC is $C_{2L}^{2} = L*(2L - 1)$,$$FC = \left( {\begin{array}{*{20}l} {FC_{1} } & {FC_{2} } & {FC_{3} } & {...} & {FC_{L(2L - 1)} } \\ \end{array} } \right)$$.
(5) Calculate the 256 bit hash value of FC to obtain the feature code SK of the carrier image
  7 $$SK = Hash - {256}\left( {FC} \right)$$

### Integer wavelet transform

**Figure 4:**
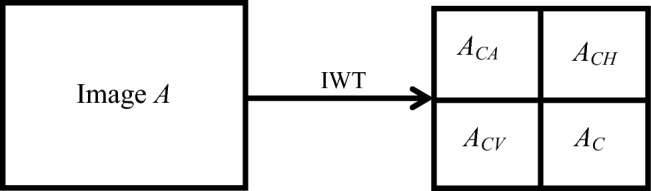
The integer wavelet transform (IWT) of image A.

The pixel values of digital images are represented by positive integers, however, the data obtained from wavelet transform of digital images is no longer integers. The accuracy of binary representation for floating-point numbers is limited, so there may be energy loss when reconstructing the obtained wavelet coefficients. The emergence of integer wavelets perfectly solves this problem. Integer wavelet transform (IWT) and inverse integer wavelet transform (IIWT) are completely reversible, which can achieve integer to integer wavelet transform. Perform IWT operation on image *A* to obtain low-frequency components *A*_*CA*_ and high-frequency components *A*_*CH*_*, A*_*CV*_, and *A*_*CD*_, as shown in Fig. 4. In the embedding stage, the intermediate ciphertext image will be embedded into the high-frequency component of the integer wavelet transform of the carrier image.

### Construction of Gaussian measurement matrix in compressed sensing (CS)

Compressed sensing is the process of compressing data while collecting signals, with the aim of collecting as few signals as possible and reconstructing the original signal with high probability^26^.

For an original signal *x*($x \in R^{n}$), assuming its coefficient vector under a set of orthogonal bases $\psi$ is *S*, the signal can be represented as(8)

8 $$x = \psi s$$

Among them, $\psi$ is sparse base or sparse dictionary.* S* is a vector composed of weighted coefficients, called a sparse representation of* x*.So the measurement process of signal *x* can be expressed as (9)

9 $$y = \varphi x = \varphi \psi s = \theta s$$

Among them, $\psi$ is the measurement matrix that projects the high-dimensional signal *x* onto a low dimensional space.The signal recovery process involves obtaining *S* from the measured value *y*, and then obtaining the original signal* x* from $x = \psi s$.When the measurement matrix satisfies the finite isometry property(RIP), the signal *x* can be accurately reconstructed, and commonly used reconstruction algorithms include: matching pursuit (MP), orthogonal matching pursuit (OMP), smooth* l*_0_ norm (*SL*_0_)^27^.

In the theory of compressed sensing, signal acquisition, compressed measurement, and reconstruction largely rely on the measurement matrix, so the measurement matrix plays an important role. The Gaussian random measurement matrix has significant randomness, can accurately reconstruct the signal through a small number of measurement values, which is widely used. A Gaussian measurement matrix was constructed based on Chebyshev map, which is defined as (10)

10 $$y(i + 1) = \cos \left( {4 \times \arccos (y(i))} \right)$$

The probability density function of the chaotic sequence generated by Chebyshev map is shown as (11)

11 $$\rho _{Y} (y) = \left\{ {\begin{array}{*{20}l} {\frac{1}{{\pi \sqrt {1 - x^{2} } }};} & {if\; - 1 < x < 1} \\ {0;} & {else} \\ \end{array} } \right.$$

The detailed construction steps of the Gaussian measurement matrix is as follows.

Step 1: Extracts the feature code *SK* of the carrier image, then divide the 256 bits *SK* into 32 groups in groups of 8 bits. Therefore, *SK* can be represented as *SK* = *sk*_1_,*sk*_2_,*sk*_3_,…,*sk*_32_ (*sk*_*i*_ is an 8-bit binary bit composed of 0 and 1). The two initial values *y*_1_(0), *y*_2_(0) of Chebyshev map are generated according to Eqs. (12)–(17):

12 $$h_{1} = \frac{{sk_{1} \oplus sk_{2} \oplus sk_{3} \oplus sk_{4} \oplus sk_{5} \oplus sk_{6} \oplus sk_{7} \oplus sk_{8} }}{{256}}$$

13 $$h_{2} = \frac{{sk_{9} \oplus sk_{10} \oplus sk_{11} \oplus sk_{12} \oplus sk_{13} \oplus sk_{14} \oplus sk_{15} \oplus sk_{16} }}{256}$$

14 $$h_{3} = \frac{{sk_{17} \oplus sk_{18} \oplus sk_{19} \oplus sk_{20} \oplus sk_{21} \oplus sk_{22} \oplus sk_{23} \oplus sk_{24} }}{256}$$

15 $$h_{4} = \frac{{sk_{{25}} \oplus sk_{{26}} \oplus sk_{{27}} \oplus sk_{{28}} \oplus sk_{{29}} \oplus sk_{{30}} \oplus sk_{{31}} \oplus sk_{{32}} }}{{256}}$$

16 $$y_{1} \left( 0 \right) = y_{1}^{\prime} \left( 0 \right) + \frac{1}{2}(h_{1} + h_{2} )$$

17 $$y_{2} \left( 0 \right) = y_{2}^{\prime} \left( 0 \right) + \frac{1}{2}(h_{3} + h_{4} )$$

where $y_{1}^{\prime} \left( 0 \right),y_{2}^{\prime} \left( 0 \right)$ are the given initial values.

Step 2: Set two initial values $\left[ {ty,sy} \right] = sort\left( {Y_{1} } \right)$ separately and iterate Chebyshev map to generate two chaotic sequences *Y*_1_ and *Y*_2_ with length *L* = *M* × *N*, respectively. Transform the chaotic sequences *Y*_1_ and* Y*_2_ to obtain two sequences *D*_1_ and *D*_2_ by formulas (18) and (19)

18 $$D_{1} = 2\left| {\frac{1}{\pi }\arcsin (Y_{1} )} \right|$$

19 $$D_{2} = 2\left| {\frac{1}{\pi }\arcsin (Y_{2} )} \right|$$

As pointed out in^28^,* D*_1_ and* D*_2_ obey the uniform distribution on the interval (0, 1).

Step 3: Using Box-Muller transformation^29^ to transform *D*_1_ and *D*_2_ into random sequences *Z*_1_ and *Z*_2_ that obey the standard Gaussian distribution according to formulas (20) and (21).

20 $$Z_{1} = \sqrt { - 2\ln \left( {D_{1} } \right) \times \cos \left( {2\pi D_{2} } \right)}$$

21 $$Z_{2} = \sqrt { - 2\ln \left( {D_{1} } \right) \times \sin \left( {2\pi D_{2} } \right)}$$

Then, the transformation of the two sequence Z_1_ Z_2_ into two matrixs $\left[ {ty,sy} \right] = sort\left( {Y_{1} } \right)$, and $\left[ {ty,sy} \right] = sort\left( {Y_{1} } \right)$,which is the Gauss measurement matrix. But the optimized Gaussian measurement matrix can reduce the influence of signal noise and improve the quality of reconstructed image^30^.

Step 4: Perform singular value decomposition(SVD) on matrix $\left[ {ty,sy} \right] = sort\left( {Y_{1} } \right)$ as $\left[ {ty,sy} \right] = sort\left( {Y_{1} } \right)$. Where, $\left[ {ty,sy} \right] = sort\left( {Y_{1} } \right)$ is a diagonal matrix as: $\left[ {ty,sy} \right] = sort\left( {Y_{1} } \right)$. Calculate the mean value of principal diagonal elements of $\left[ {ty,sy} \right] = sort\left( {Y_{1} } \right)$ as $\left[ {ty,sy} \right] = sort\left( {Y_{1} } \right)$.

Define $\left[ {ty,sy} \right] = sort\left( {Y_{1} } \right)$, in which $\left[ {ty,sy} \right] = sort\left( {Y_{1} } \right)$ and construct the optimized matrix $\left[ {ty,sy} \right] = sort\left( {Y_{1} } \right)$ as formula (22).

22 $$\bar{\psi_{1} }^{ - } = U\left( {\begin{array}{*{20}l} {\mathop \sum \limits^{ - } } & 0 \\ 0 & 0 \\ \end{array} } \right)V^{T}$$

By optimizing $\left[ {ty,sy} \right] = sort\left( {Y_{1} } \right)$ using the same method, we can obtain $\left[ {ty,sy} \right] = sort\left( {Y_{1} } \right)$.

Then, the new matrix $\left[ {ty,sy} \right] = sort\left( {Y_{1} } \right)$ and $\left[ {ty,sy} \right] = sort\left( {Y_{1} } \right)$ are the Gaussian measurement matrices that will be used in encryption algorithm.

## Encryption algorithm

**Figure 5:**
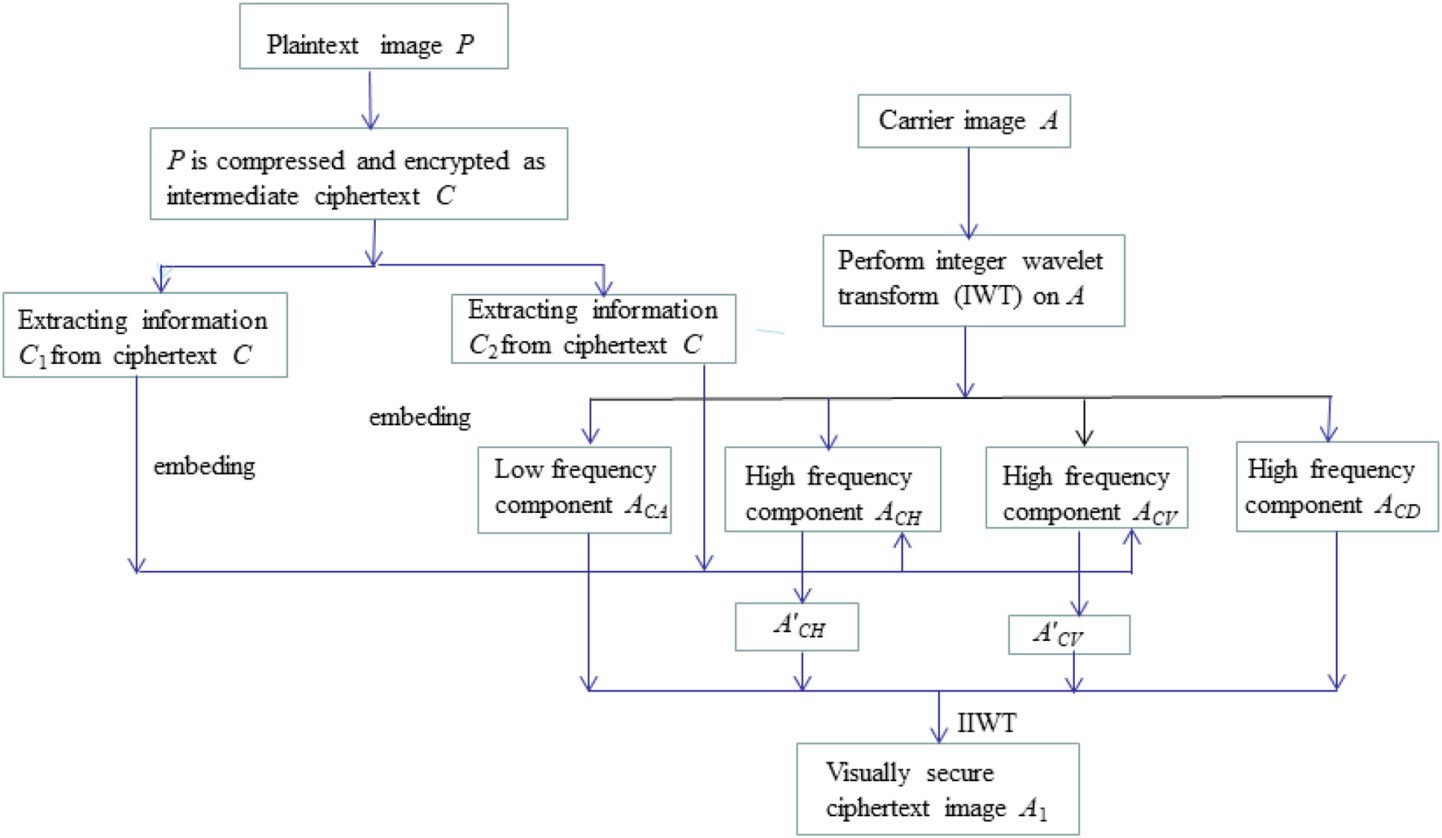
The overall flowchart of the proposed encryption algorithm.

The proposed encryption scheme consists of two stages. In the first stage, the plaintext image is compressed and encrypted into a ciphertext image, called intermediate ciphertext.In the second stage, the intermediate ciphertext is embeded into the high-frequency part of the integer wavelet transform domain of the carrier image, and then performs inverse transformation on the carrier image to obtain the corresponding visual security ciphertext image,called ciphertext. The flowchart of the proposed encryption algorithm is shown in Fig. 5.

### Compression and encryption algorithm for the plaintext image

**Figure 6:**
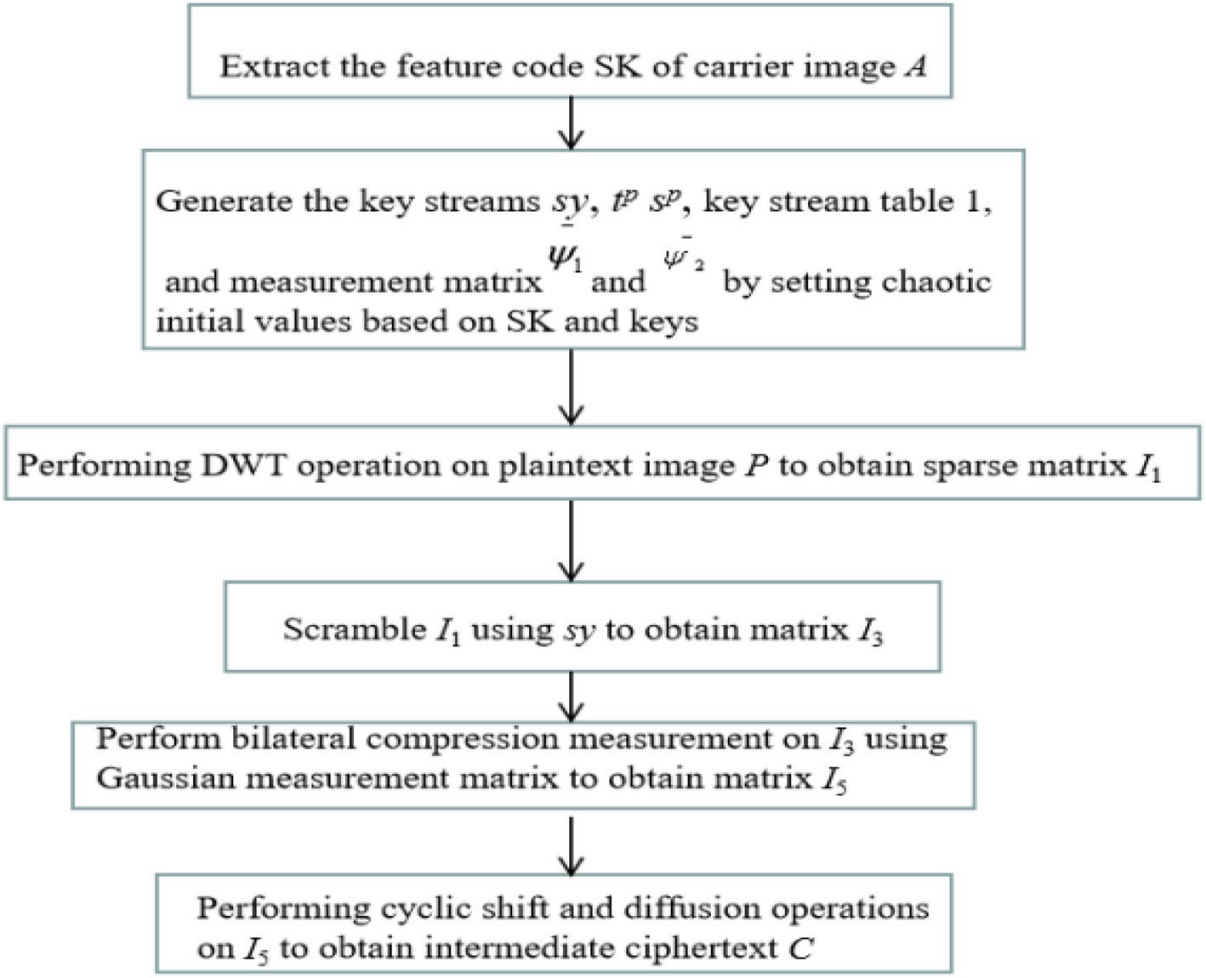
Compression encryption algorithm flowchart.

In the first stage, the plaintext image is compressed and encrypted into intermediate ciphertext. The algorithm flowchart is shown in Fig. 6.

The detailed steps of the algorithm are as follows:

Step 1: Implement two-dimensional discrete wavelet transform (2-D DWT) on the grayscale plaintext image I of the size *M* × *N* for sparsity.And set the threshold TS, so that the absolute value of elements in the sparse matrix that are less than TS becomes 0, ultimately,we can obtain the sparse matrix* I*_1_ with the size o*f M* × *N.*

Step 2: Arrange the chaotic sequence Y_1_ generated by the method in Sect. 1.6 in ascending order as formula (23).

23 $$\left[ {ty,sy} \right] = sort\left( {Y_{1} } \right)$$

Among them, *ty* is a new sequence obtained by arranging *Y*_1_ in ascending order, and *sy* is also a new sequence formed by the positional index of *ty* in *Y*_1_.

Convert the sparse matrix *I*_1_ into a one-dimensional vector *I*_2_ with a length of* M* * *N*, and then perform a scrambling operation on *I*_2_ according to *sy* to obtain *I*_3._Then convert* I*_3_ into matrix *I*_4_ with the size o*f M* × *N.* as formula (24).

24 $$I_{3} \left( i \right) = I_{2} \left( {sy\left( i \right)} \right)$$

Step 3: Use the measurement matrixes $\bar{\psi }_{1} \bar{\psi }_{2}$ to perform bidirectional two-dimensional compressed sensing (2-DCS) on the sparse matrix *I*_4_ obtained in Step 2, as shown in formula (25)

25 $$I_{5} = {\bar{\psi}}^{ - }_{1} I_{4} {\bar{\psi}}_{2}^{T}$$

*I*_5_ is the compression measurement result of* I*_4_, with a size of *M*_1_ × *N*_1_, where *M*_1_ = *M* × *CR*, *N*_1_ = *N* × *CR*, *CR* is the set compression rate.

Step 4: The element values in matrix I_5_ are relatively large, and the magnitude can reach 10^3^, the range of element values can be adjusted to [0 255] by the following transformation of (26), so* I*_6_ is the compressed image.

26 $$I_{6} = {\text{round}}((I_{5} - min) \times 255/(max - min))$$

where, *max* and *min* are the maximum and minimum values of *I*5 respectively. And round(*X*) rounds each element of *X* to the nearest integer.

Step 5:According to Eq. (27), the sequence *TP*={ *tp*(1), *tp*(2), *tp*(3),…,* tp*(*l*) }, *tp*(*i*)∈[0,7] (*i*=1, 2, 3, …, *l*) is generated using *Y*1. Similarly, perform the operation on sequence *Y*2 to obtain sequence *SP*={ *sp*(1), *sp*(2), *sp*(3),…, *sp*(*l*) }, *sp*(*i*)∈[0,5], (*i*=1, 2, 3, …,* l*) according to Eq. (28).

27 $$t^{p} \left( i \right) = \bmod \left( {floor\left( {y_{1} \left( i \right) \times 10^{12} } \right),8} \right)$$

28 $$s^{p} \left( i \right) = \bmod \left( {floor\left( {y_{2} \left( i \right) \times 10^{12} } \right),6} \right)$$

Step 6: Convert *I*6 into a one-dimensional vector *I*7 with a length of* l*=*M*1 * *N*1, perform a cyclic shift operation on *I*7, and the cyclic shift bits of different pixels are determined based on the value of *TP*. Then perform diffusion operation to confuse the relationship between ciphertext and plaintext and *SP* (*i*) determines the selection of different diffusion key streams from Table 1.

For example, if *s*^*p*^ (1) = 3, then *Key*_1_ (1) = *T*_1,4,5_ (1), *Key*_2_ (1) = *T*_3,4,6_ (1). *T*_1,4,5_ (1) and *T*_3,4,6_ (1) are used for diffusion operations on *c*(1).

The final cyclic shift formula and ciphertext diffusion formula are the following Eqs. (29)–(31), resulting in the ciphertext sequence ***C*** = {*c*(1), *c*(2), *c*(3), …, *c*(*l*)}. Convert C to a matrix with a size of *M*_1_ × *N*_1_,to obtain the intermediate ciphertext.

29 $$I_{8} \left( i \right) = circshift(I_{7} \left( i \right),t^{p} \left( i \right))\quad$$

30 $$c\left( 0 \right) = \bmod \left( {floor\left( {y_{2} \left( 1 \right) \times 10^{12} } \right),256} \right)$$

31 $$c\left( i \right) = I_{8} \left( i \right) \oplus Key_{2} \left( i \right) \oplus \left( {\bmod \left( {\left( {c\left( {i - 1} \right) + Key_{1} \left( i \right)} \right),256} \right)} \right),\quad i = 2,3, \ldots ,l$$

Due to the correlation between the generation of *Y*_1_, *Y*_2_ and the feature codes of the carrier image, the key streams *T*^*P*^ and *S*^*P*^ are both related to the carrier image, which further enhances the security of the encryption system.

### Embedding compressed ciphertext images into carrier images

Step 1: Implement integer wavelet transform(IWT) on carrier image A with the size of *M* × *N* to obtain low-frequency components *A*_*CA*_ and high-frequency components* A*_*CH*_, *A*_*CV*_, and* A*_*CD*_, all of which are (*M*/2) × (*N*/2).

Step 2: In order to perfectly embed the ciphertext image *C* into the carrier image, we set the compression rate *CR* of the plaintext image to *CR* = 0.5, so that the size of the ciphertext image* C* is (*M*/2) × (*N*/2). In order to securely embed matrix *C* into the carrier image, matrices *C*_1_ and *C*_2_ can be obtained according to the formulas (32) and (33).

32 $$C_{1} = floor(C/10)$$

33 $$C_{2} = \bmod (C,10)$$

Step 3: Replace the high-frequency component matrices *A*_*CH*_ and *A*_*CV*_ with the matrices *C*_1_ and *C*_2_ to obtain the matrices* A*_*CH*_ ' and *A*_*CV*_'. Perform inverse integer wavelet transform on matrices *A*_*CA*_, *A*_*CH*_ ', *A*_*CV*_' and *A*_*CD*_ to obtain matrix *A*_1_, which is a visually secure ciphertext image, called ciphertext.

## Image decryption algorithm

The decryption algorithm is the inverse operation of the encryption algorithm. It consists of two stages: In the first stage, the intermediate ciphertext is extracted from the ciphertext; In the second stage, the plaintext image* I* is restored from the intermediate ciphertext.

### Extracting the intermediate ciphertext from the ciphertext

Step 1: Implement integer wavelet transform (IWT) on the ciphertext *A*_1_ with the size of *M* × *N* to obtain low-frequency components *A*_1*CA*_ and high-frequency components* A*_1*CH*_, *A*_1*CV*_, and* A*_1*CD*_, all of which are (*M*/2) × (*N*/2).

Step 2: Extract the information of matrices* A*_1*CH*_ and* A*_1*CV*_, and record them as *C*_1_ and *C*_2_ respectively. So the intermediate ciphertext image *C* can be obtained through the formula (34)

34 $${\text{C}} = {1}0C_{{1}} + C_{{2}}$$

### Reconstruction of plaintext images from the intermediate ciphertext

Any perfect encryption algorithm should be decrypted by someone with the correct key, that is, the algorithm is reversible. Otherwise, such encryption algorithms are meaningless. The decryption process is the reverse of the encryption process, and the specific steps are as follows:

Step 1: The receiver receives the ciphertext image and extracts the feature code SK according to the method in Sect. 1.3. Due to the robustness of the feature code, the extracted feature code is exactly the same as the feature code of the original carrier image.

Step 2: Set the initial value of the Chebyshev map according to the given key and the extracted feature code SK to generate the measurement matrixes ${\bar{\psi}_{1} }^{ - }$, ${\bar{\psi}_{2} }^{ - }$ and two random sequences *T*^***P***^ = {* t*^*p*^(1),* t*^*p*^(2),* t*^*p*^(3),…,* t*^*p*^(*l*) },* t*^*p*^(*i*) ∈ [0,7] and ***S***^***P***^ = {* s*^*p*^(1),* s*^*p*^(2),* s*^*p*^(3),…,* s*^*p*^(*l*) },* s*^*p*^(*i*) ∈ [0,5].

Step 3: According to the given keys, set the initial value of the four-dimensional chaotic system to iterate the hyperchaotic system (1), and generate the key flows in Table 1.

Step 4: *Key*_1_ (*i*) and *Key*_2_ (*i*) can be determined by *s*^*p*^(*i*), thereby *I*_8_(*i*) can be decrypted according to the formula (35). Furthermore, *I*_7_ (*i*) can be decrypted according to Eq. (36).

35 $$I_{8} \left( i \right) = c\left( i \right) \oplus key_{2} \left( i \right) \oplus (\bmod ((c\left( {i - 1} \right) + key_{1} \left( i \right)),256))$$

36 $$I_{7} (i) = circshift\left( {I_{8} (i), - t^{p} (i)} \right)\quad i = 1,2,3, \ldots M^{*} N/4$$

Step 5: According to the formula (37), recover* I*_6_ from *I*_7_ and convert *I*_6_ to the matrix *I*_5_ of the size *M*_1_ × *N*_1_

37 $$I_{6} = I_{7} \times (max - min)/255 + min$$

Step 6: The OMP algorithm is used to reconstruct the signal *I*_4_ from *I*_5_ according to the measurement matrix $\bar{\psi}^{ - }_{1} \bar{\psi}^{ - }_{2}$.

Step 7: Convert* I*_4_ into a one-dimensional vector* I*_3_ with a length of* M* * *N*. Use *sy* to scramble *I*_3_ to obtain *I*_2_, and then convert *I*_2_ to the matrix *I*_1_ of the size *M* × *N* .Finally, perform inverse wavelet transform on* I*_1_ to obtain the final plaintext image *I*.

### Experimental results and performance analysis

Next, the experimental simulation of the encryption algorithm is carried out, and the uniqueness and robustness of the feature codes are further verified through experiments.

## Experimental results

**Figure 7:**
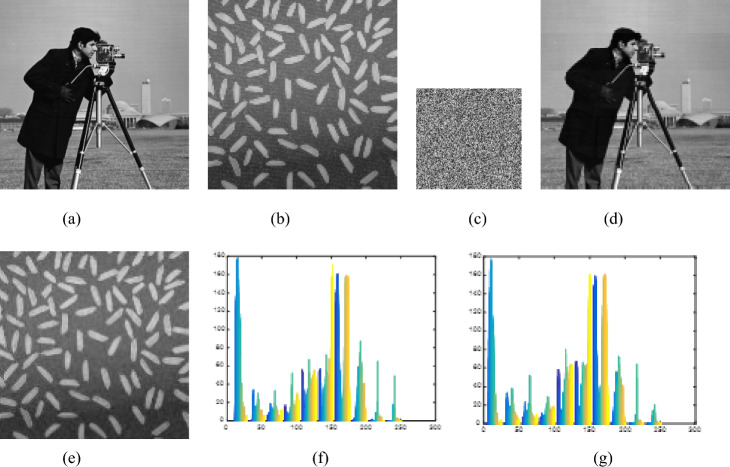
The encryption and decryption results. (**a**) The Carrier image. (**b**) The plaintext image. (**c**) The intermediate ciphertext. (**d**) The ciphertext image. (**e**) The decrypted image. (**f**) The histogram of (**a**). (**g**) The histogram of (**d**).

Experiments were conducted on a PC configured with an Intel (R) Core (TM) i5-9400F CPU running at 2.90 GHz with 16 GB memory and a Windows 10 64-bit operating system. The above encryption algorithm was implemented using MATLAB R2014a. We choose the image cameraman as the carrier image, and the image rice as the plaintext image, both of which are 256 × 256 in size. As long as the plaintext image is smaller than the carrier image, good hiding effects can be achieved. In the experiment, the parameter settings are as follows: The initial values of the Chebyshev map are set to $y_{1}^{\prime} \left( 0 \right) = 0.3468y_{2}^{\prime} \left( 0 \right) = 0.6821$ and the initial values of the new four-dimensional chaos are set to *x*_1_(0) = 0.9654, *x*_2_(0) = 0.0546, *x*_3_(0) = 0.6705, *x*_4_(0) = 0.5698. Set the threshold TS = 30 for plaintext sparsity and compression rate CR = 0.5. The plaintext image is compressed and encrypted to become an intermediate ciphertext image, with a size of 128 × 128, which is exactly hidden in the carrier image When extracting the feature code of the carrier image, set *n* = 34, *m* = 50. Therefore, the length of the obtained feature code FC is 153. The OMP method is adopted in the reconstruction process of CS. The encryption and decryption results are shown in Fig. 7.

From Fig. 7, it can be seen that: (1) Fig. 7a,b are the carrier image and plaintext image, respectively and Fig. 7c is the intermediate ciphertext image. When it is transmitted and stored on the network, it is easily discovered and attacked by hackers, and cannot protect the security of plaintext image data. (2) Fig. 7d is the final grayscale visually secure ciphertext image, which is visually meaningful and will not be considered as a ciphertext image from the appearance. It can effectively hide ciphertext information and has a relatively high level of appearance security. (3) The histogram of carrier image (a) is almost identical to that of the ciphertext image (d). (4) The decrypted image shown in (e) is visually almost identical to the plaintext image shown in Fig. 7b, indicating that the reconstruction quality of the image is satisfactory.

Peak Signal to Noise Ratio (PSNR) is the most common objective method for evaluating the similarity between the ciphertext image and the carrier image^31^. A larger PSNR value indicates a smaller difference between the two images, resulting in relatively high level of appearance security. The calculation formula for PSNR is as shown in formulas (38) and (39).

38 $$PSNR = 10\log_{10} \frac{{255^{2} }}{{\sqrt {MSE} }}$$

39 $$MSE = \frac{1}{M*N}\sum\limits_{i = 1}^{M} {\sum\limits_{j = 1}^{N} {\left( {X\left( {i,j} \right) - Y\left( {i,j} \right)} \right)} }^{2}$$

Among them, *M* and* N* represent the number of rows and columns of the image, while *X*(*i*,* j*) and *Y* (*i*, *j*) represent the pixel values of the ciphertext image *X* and the Carrier image *Y* at positions (*i*, *j*), respectively. Table 3 presents the PSNR values between different carrier images and corresponding visual security ciphertext images, as well as the comparative analysis results with other literature algorithms^32–34^. It can be seen that the PSNR values in this algorithm are higher than those of the other three algorithms, indicating that the embedding method has little impact on the carrier image. Therefore, visually, there is almost no difference between the ciphertext image and the carrier image, and the similarity is very high.

**Table 3:** Comparison of PNSR between this algorithm and other algorithms.

Algorithms	The plaintext images	The carrier images	PSNR(dB)
Reference^32^	Rice	Cameraman	35.23
		Peppers	34.12
		kids	33.25
		pears	32.68
		trees	33.17
Reference^33^	Rice	Cameraman	32.18
		Peppers	27.89
		kids	29.97
		pears	30.16
		trees	29.98
Reference^34^	Rice	Cameraman	34.23
		Peppers	32.87
		kids	31.02
		pears	31.97
		trees	32.31
Our algorithm	Rice	Cameraman	35.87
		Peppers	34.82
		kids	33.79
		pears	33.18
		trees	32.74

### Analysis of the effectiveness of carrier image feature codes

The effectiveness analysis of carrier image feature codes mainly includes robustness and uniqueness analysis. Robustness refers to the feature codes of the original carrier image, the visual security ciphertext image feature codes, and the attacked ciphertext image feature codes, all of which must be the same. Uniqueness refers to the fact that the feature codes should be different for different images. Table 4 shows the feature codes of different carrier images and corresponding ciphertext images. From Table 4, it can be seen that (1) the feature codes have uniqueness, and the feature codes of different images are different; (2) The feature code is robust and remains unchanged after being embedded with secret information.

**Table 4:** Feature codes of different carrier images and corresponding ciphertext images.

Carrier images	Feature codes of carrier images	Feature codes of ciphertext images
Cameraman	7d3c483c187ed8bfd66822e3740d5885a9f782b038382f458874ab9679b1a5e4	7d3c483c187ed8bfd66822e3740d5885a9f782b038382f458874ab9679b1a5e4
Peppers	e18e1cbda7d1db618df98d3101f34910635d373d5cc80e827faa78b611781080	e18e1cbda7d1db618df98d3101f34910635d373d5cc80e827faa78b611781080
Kids	65a85e6d36fc8859488fdbedbbe75597c03d35e4ec434e0e40e4e62158eb037c	65a85e6d36fc8859488fdbedbbe75597c03d35e4ec434e0e40e4e62158eb037c
Pears	3dfefd8140e11f40d4db8587476d09b05401b4b938ff7c86b6350768388d1005	3dfefd8140e11f40d4db8587476d09b05401b4b938ff7c86b6350768388d1005
Trees	c4cbe32f209e0e4e915110dcccb401d0200d3c77bacfa9fb6106eb0062c02f22	c4cbe32f209e0e4e915110dcccb401d0200d3c77bacfa9fb6106eb0062c02f22

In addition, the feature codes of the ciphertext image can still remain unchanged after being contaminated by various types of noise. In addition, the feature codes of ciphertext images can still remain unchanged after being contaminated by various types of noise. Tables 5, 6 and 7 respectively show that the feature codes extracted from “cameraman” ciphertext images are unchanged after being contaminated by varying degrees of pepper and salt noise (SPN), Gaussian noise (GN), and speckle noise (SN).Similarly, using “Peppers,” “kids,” “ears,” and “trees” as carrier images, the corresponding ciphertext images are obtained by embedding secret information. The feature codes of these ciphertext images remain unchanged even after being contaminated with varying degrees of noise.

**Table 5:** The feature codes of ciphertext images attacked by varying degrees of salt and pepper noise.

Contaminated degrees	0.000005% SPN	0.000007% SPN	0.000009% SPN	0.00001% SPN
Is the feature code synchronized?	Yes	Yes	Yes	Yes

**Table 6:** The feature codes of ciphertext images attacked by varying degrees of Gaussian noise.

Contaminated degrees	0.000001% GN	0.000003% GN	0.000005% GN	0.000007% GN
Is the feature code synchronized?	Yes	Yes	Yes	Yes

**Table 7:** The feature codes of ciphertext images attacked by varying degrees of speckle noise.

Contaminated degrees	0.000001% SN	0.000003% SN	0.000005% SN	0.000007% SN
Is the feature code synchronized?	Yes	Yes	Yes	Yes

## Safety analysis

This chapter analyzes the security of intermediate ciphertext from the aspects of key space, key sensitivity, correlation coefficient, information entropy, and resistance to noise attacks,

### Key space analysis

All the keys available during encryption are the key space, and the larger the key space, the better the performance of resisting brute force attacks. The key set of this algorithm includes the initial values {*x*_1_(0), *x*_2_(0), *x*_3_(0), *x*_4_(0)}of the four-dimensional chaotic system and the initial values of the Chebyshev map $y_{1}^{\prime} \left( 0 \right)y_{2}^{\prime} \left( 0 \right)$. Experimental verification shows that the accuracy of *x*_1_(0), *x*_2_(0), *x*_3_(0), *x*_4_(0) can reach 10^–15^, and the accuracy of $y_{1}^{\prime} \left( 0 \right)y_{2}^{\prime} \left( 0 \right)$ can reach 10^–14^. Therefore, the key space of the encryption system can reach 10^15^ × 10^15^ × 10^15^ × 10^15^ × 10^14^ × 10^14^ = 10^84^ ≈ 2^279^. In addition, the threshold *TS* can also be used as an encryption key. So the key space is much larger than the key space of a secure cryptographic system pointed out in reference^35^, which should be greater than 2^100^. Therefore, our algorithm is secure against brute force attacks.

### Key sensitivity analysis

**Figure 8:**
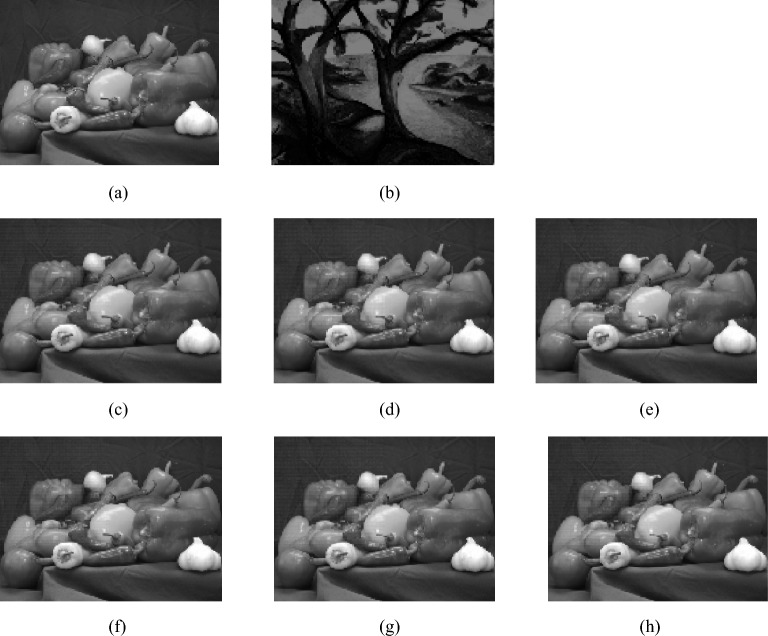
The impact of modifying encryption keys on encryption results. (**a**) The carrier image. (**b**) The Plaintext image. (**c**) Encryption result of key keys1. (**d**) Encryption result of key keys2. (**e**) Encryption result of key keys3. (**f**) Encryption result of key keys4. (**g**) Encryption result of key keys5. (**h**) Encryption result of key keys6.

In this section, image ‘Trees’ is used as plaintext images, as shown in Fig. 8a, and image ‘Peppers’ is used as carrier images, as shown in Fig. 8b. We will test both encryption key sensitivity and decryption key sensitivity.

1. Sensitivity analysis of encryption keys

Firstly, the sensitivity of the encryption keys are tested. The selected keys is keys1- keys6 in Table 8 and the encryption results of different keys are shown in Fig. 8c–h.

**Table 8:** The error keys used in decryption.

Keys	*x*_1_(0)	*x*_2_(0)	*x*_3_(0)	*x*_4_(0)	$y_{1}^{\prime} \left( 0 \right)$	*y*′_2_ (0)
Keys1	0.9654 + 10^−15^	0.0546	0.6705	0.5698	0.3468	0.6821
Keys2	0.9654	0.0546 + 10^−15^	0.6705	0.5698	0.3468	0.6821
Keys3	0.9654	0.0546	0.6705 + 10^−15^	0.5698	0.3468	0.6821
Keys4	0.9654	0.0546	0.6705	0.5698 + 10^−15^	0.3468	0.6821
Keys5	0.9654	0.0546	0.6705	0.5698	0.3468 + 10^−14^	0.6821
Keys6	0.9654	0.0546	0.6705	0.5698	0.3468	0.6821 + 10^−14^

From Fig. 8, it can be seen that when the key is changed, there is no significant visual difference in the ciphertext image. This is because the carrier image plays an important role in visual security, so the modification of the encryption key has little impact on the encryption results.

- (2) Sensitivity analysis of decryption keys

**Figure 9:**
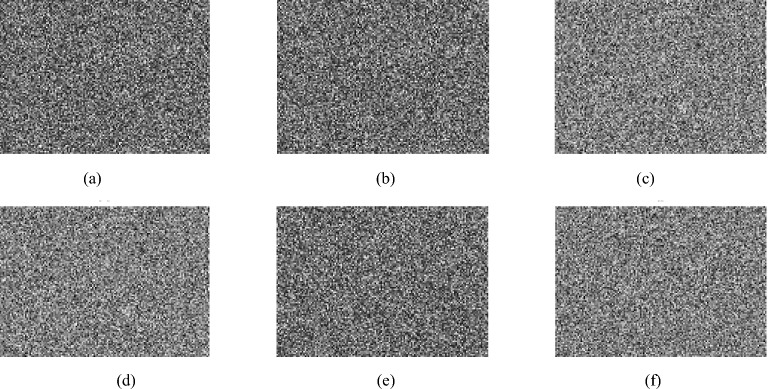
The decrypted images by using the error keys. (**a**) The decrypted image by Keys1. (**b**) The decrypted image by Keys2. (**c**) The decrypted image by Keys3. (**d**) The decrypted image by Keys4. (**e**) The decrypted image by Keys5. (**f**) The decrypted image by Keys6.

A secure encryption algorithm should be sensitive to keys in order to resist brute force attacks. The algorithm is sensitive to keys, which means that even if there is a slight difference between the decryption key and the correct key, no information about the plaintext image can be obtained during decryption. Using‘trees’ image as the plaintext image and ‘Peppers’ image as the carrier image, the initial values of Chebyshev map are set to $y_{1}^{\prime} \left( 0 \right) = 0.3468y_{2}^{\prime} \left( 0 \right) = 0.6821$ and the initial values of the new four-dimensional chaos are set to *x*_1_(0) = 0.9654, *x*_2_(0) = 0.0546, *x*_3_(0) = 0.6705, *x*_4_(0) = 0.5698, with a threshold of TS = 30. The encryption operation is performed to obtain the ciphertext image, while the decryption results are shown in the Fig. 9 using Keys1~Keys6 in Table 8.

In Fig. 9a–f represent the decryption results with the error keys in Table 8, indicating that no information about the plaintext image can be obtained. This further demonstrates the high sensitivity of the algorithm to keys.

### Correlation coefficient analysis

For a natural image, adjacent pixels have strong correlation and redundancy. One of the goals of image encryption is to eliminate this redundancy and reduce the correlation between adjacent pixels. For the plaintext image and ciphertext image to be evaluated, 4000 pixel points are randomly selected as reference points. Based on these points, adjacent pixel points are taken along the horizontal, vertical, and diagonal directions to form pixel pairs. The correlation coefficient values of the plaintext image,the intermediate ciphertext and the ciphertext image in these three directions are calculated by using the correlation coefficient formula (40), and the calculation results are shown in Table 9.

40 $$xc = \frac{{n\sum\limits_{i = 1}^{n} {x_{i} y_{i} - \sum\limits_{i = 1}^{n} {x_{i} \sum\limits_{i = 1}^{n} {y_{i} } } } }}{{\sqrt {n\sum\limits_{i = 1}^{n} {x_{i}^{2} - \left( {\sum\limits_{i = 1}^{n} {x_{i} } } \right)^{2} \sqrt {n\sum\limits_{i = 1}^{n} {y_{i}^{2} - \left( {\sum\limits_{i = 1}^{n} {y_{i} } } \right)^{2} } } } } }}$$

**Table 9:** Calculation results of correlation coefficient.

Images	Horizontal	Vertical	Diagonal
“Rice” Plaintext	0.9427	0.9263	0.8994
“Rice” intermediate ciphertext	− 0.0026	0.0127	− 0.0261
“Cameraman” Carrier image	0.9588	0.9360	0.9095
“Cameraman” ciphertext image	0.9413	0.9189	0.8876
“autumn” Plaintext	0.9675	0.9845	0.9821
“autumn” intermediate ciphertext	− 0.0056	0.0132	0.0109
“peppers” Carrier image	0.9894	0.9931	0.9847
“peppers” ciphertext image	0.9817	0.9912	0.9821

Among them, *x*_*i*_ and *y*_*i*_ represent the grayscale values of adjacent two pixels, and* n* represents the number of selected pixel pairs.

It can be seen from Table 9 that: (1) The adjacent pixels of plaintext images, carrier images, and ciphertext images have high correlation in all directions, with strong correlation coefficients greater than 0.9. This indicates that although plaintext information data is embedded into the carrier image, the information of the carrier image is still retained, ensuring the visual security of the obtained ciphertext image. (2) The intermediate ciphertext image has weak correlation between adjacent pixels, and the correlation coefficient has significantly decreased, almost equal to 0.

In addition, Table 10 presents the comparison results of the correlation coefficients of different images calculated by our algorithm and literature^36–38^. The comparison results show that the adjacent pixel correlation of the intermediate ciphertext image obtained by our algorithm is small, which is better than these three literatures.

**Table 10:** Comparison results of image correlation coefficients for different algorithms.

The algorithms	Images	Horizontal direction	Vertical direction	Diagonal direction
	Rice” Plaintext	0.9427	0.9263	0.8994
Ours	Rice”intermediate ciphertext	− 0.0026	0.0127	− 0.0261
Ref.^36^		0.0145	0.0356	− 0.0289
	“autumn”Plaintext	0.9675	0.9845	0.9821
Ours	autumn”intermediate ciphertext	− 0.0056	0.0132	0.0109
Ref.^37^		− 0.0098	0.0231	0.0205
Ref.^38^		− 0.0102	0.0169	0.0219

**Figure 10:**
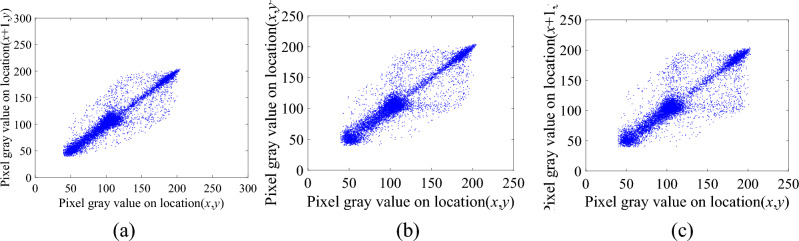
The correlation distribution of the plaintext image. (**a**) The horizontal direction. (**b**) The vertical direction. (**c**) The diagonal direction.

**Figure 11:**
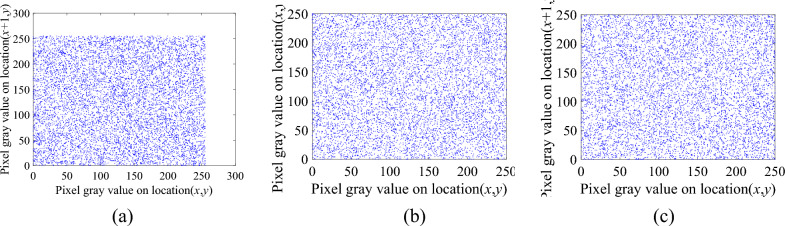
Correlation distribution map of the intermediate ciphertext image. (**a**) The horizontal direction. (**b**) The vertical direction. (**c**) The diagonal direction.

**Figure 12:**
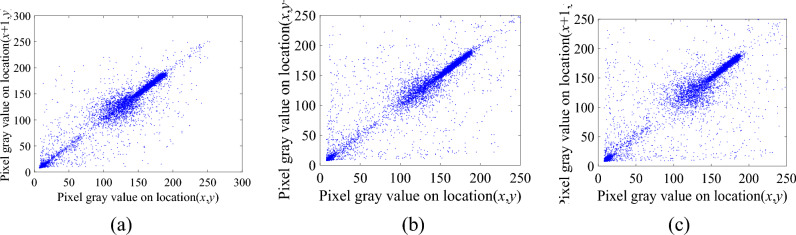
Correlation distribution map of the ciphertext image. (**a**) The horizontal direction, (**b**) The vertical direction, (**c**) The diagonal direction.

In order to compare the correlation between adjacent pixels of the plaintext image, the intermediate ciphertext image and the ciphertext image more vividly and intuitively, we use Rice as the plaintext image and cameraman as the carrier image to draw the correlation distribution maps of the plaintext image, the intermediate ciphertext image and the ciphertext image in these three directions, as shown in Figs. 10, 11 and 12, respectively.

### Information entropy analysis

The more chaotic the image, the less information it provides, resulting in a higher information entropy of the image. The calculation of information entropy is shown in formula (41)

41 $$E\left( m \right) = - \sum\limits_{i = 1}^{n - 1} {p\left( {m_{i} } \right)} \log_{2} \left( {p\left( {m_{i} } \right)} \right)$$

where *p*(*m*_*i*_) denotes the probability of gray level *m*_*i*._ Moreover, in this experiment, the variate *n* is equal to 256 and the ideal E value is 8. Under the conditions that the embedding stage is remove and the compression rate is set to 0.25, The information entropy calculation results of the plaintext image “cameraman”, the carrier image “autumn”, the intermediate ciphertext image, and the ciphertext image are shown in Table 11. It can be seen that the entropy value of the intermediate ciphertext image in the fourth column is close to 8, which can resist certain statistical attacks. In addition, the entropy value of the carrier image and its corresponding visual security ciphertext image are very close, indicating that the randomness of the carrier image is also well preserved.

**Table 11:** Information entropy of the ciphertext images.

The plaintext image cameraman	The carrier image autumn	The intermediate ciphertext image	The ciphertext image
7.0097	6.9917	7.9985	7.0149

Table 12 presents the comparison results of information entropy between our algorithm and other literature^13,37,38^ for plaintext “cameraman” image. From Table 12, it can be seen that compared with other literature^13,37,38^, the information entropy of the intermediate ciphertext image obtained using our algorithm is closer to the expected value of 8. Therefore, our algorithm is safe and reliable, and the possibility of information leakage is very small.

**Table 12:** Comparison of information entropy of ciphertext obtained using different algorithms.

Ours	Ref^37^	Ref^38^	Ref^13^
7.9985	7.9899	7.8997	7.9832

### Robustness analysis of noise attack

**Figure 13:**
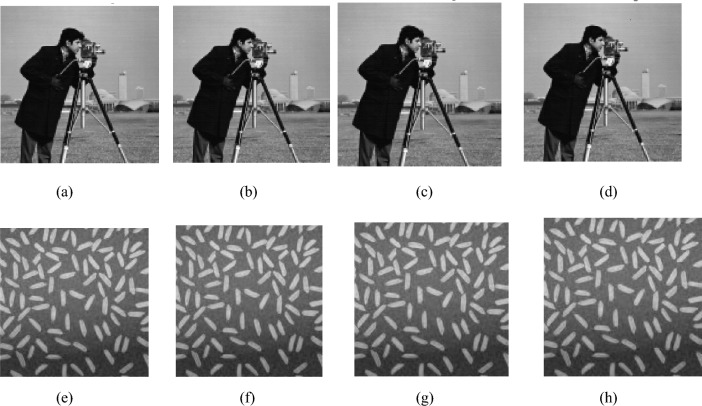
The impact of different intensities of salt and pepper noise on decryption results. (**a**) 0.000005% SPN. (**b**) 0.000007% SPN. (**c**) 0.000009% SPN. (**d**) 0.00001% SPN. (**e**) 0.000005% SPN. (**f**) 0.000007% SPN. (**g**) 0.000009% SPN. (**h**) 0.00001% SPN.

**Figure 14:**
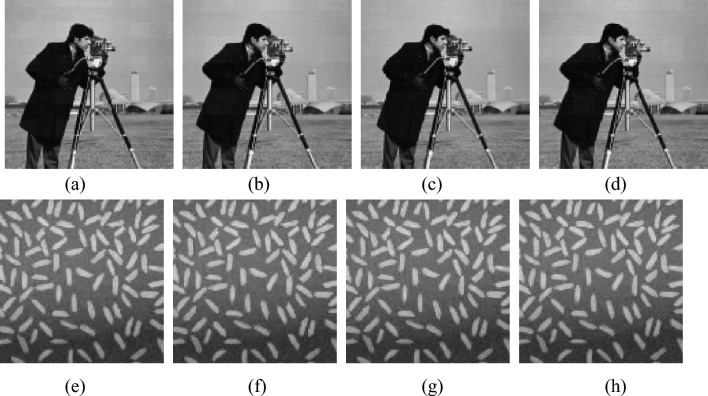
The impact of different intensities of Gaussian noise with different intensities on decryption results. (**a**) 0.000001% GN. (**b**) 0.000003% GN. (**c**) 0.000005% GN. (**d**) 0.000007% GN. (**e**) 0.000001% GN. (**f**) 0.000003% GN. (**g**) 0.000005% GN. (**h**) 0.000007% GN.

**Figure 15:**
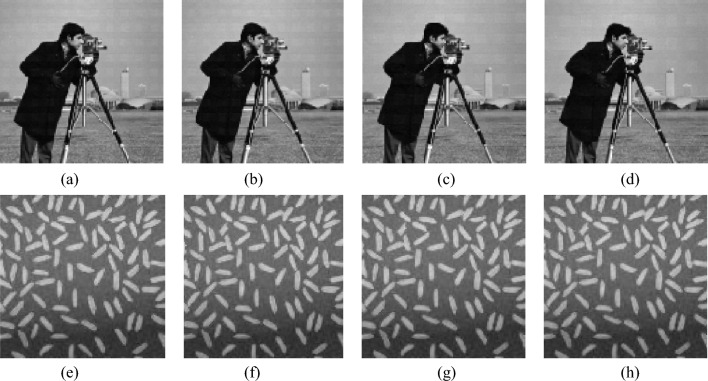
The impact of different intensities of speckle noise with different intensities on decryption results. (**a**) 0.000001% SN. (**b**) 0.000003% SN. (**c**) 0.000005% SN. (**d**) 0.000007% SN. (**e**) 0.000001% SN. (**f**) 0.000003% SN. (**g**) 0.000005% SN. (**h**) 0.000007% SN.

During network transmission, ciphertext images may be contaminated by different types of noise. These noises make it more difficult to recover plaintext images from ciphertext images, so image encryption algorithms with noise resistance are more suitable for practical applications. Therefore, we add various types of noise to visually secure ciphertext images and evaluate the ability of the proposed algorithm based on the quality of the images decrypted from the ciphertext images with various types of noise added. In this article, pepper and salt noise (SPN), Gaussian noise (GN), and speckle noise (SN) were added to the ciphertext images in Fig. 5d respectively. The other parameter settings are the same as in Sect. 4.1, and the noise intensity level and experimental results are shown in the following Figs. 13, 14 and 15.

In Fig. 13a–d are visual safety images with added intensities of 0.000005%, 0.000007%, 0.000009%, and 0.00001% salt and pepper noise (SPN), respectively; e–h are the decrypted images corresponding to a–d.

In Fig. 14, (a)–(d) are visual safety images with added intensities of 0.000001%, 0.000003%, 0.000005%, and 0.000007%, respectively; (e)–(h) are the decrypted images corresponding to (a)–(d).

In Fig. 15, (a)–(d) are visual safety images with added intensities of 0.000001%, 0.000003%, 0.000005%, and 0.000007%, respectively; (e)–(h) are the decrypted images corresponding to (a)–(d).

Usually, PSNR is used to determine the quality of decrypted images. A larger PSNR value indicates a smaller difference between the two images, resulting in higher reconstruction accuracy.

Calculate the PSNR between plaintext images and decrypted images under different intensities of noise to quantitatively evaluate the algorithm's resistance to noise, and the results are shown in Table 13.

**Table 13:** PSNR with different noise intensities.

Noise type	Noise intensity	PSNR
Salt and pepper noise SPN	0.000005%	29.5851
	0.000007%	29.5812
	0.000009%	29.4988
	0.00001%	29.4970
Gaussian noise GN	0.000001%	30.7963
	0.000003%	30.7898
	0.000005%	30.7862
	0.000007%	30.7709
Speckle noise SN	0.000001%	30.7998
	0.000003%	30.7948
	0.000005%	30.7885
	0.000007%	30.7799

From Figs. 13, 14 and 15 and Table 13, it can be seen that: (1) the decryption results of ciphertext images with adding different types of noise have little visual difference. (2) With the increase of noise intensity, the quality of the corresponding decryption results also decreases in visual quality, but there is not much difference from the original plaintext image visually.

### Time complexity analysis

Time cost is an important indicator for evaluating encryption algorithms. Table 14 presents the time cost test results for encrypting grayscale images of different sizes with a compression ratio of CR = 0.5.

**Table 14:** Encryption and decryption time for different images (time unit: s).

Size	Encryption process			Decryption process		
	Compression	Encryption	Total	Decryption	Reconstruction	Total
256 × 256	0.14298	0.27616	0.4191	0.26250	5.62098	5.8835
512 × 512	0.18992	0.33864	0.5286	0.37142	25.01365	25.3851
1024 × 1024	0.26749	0.59412	0.8916	0.60763	95.00236	95.6098

The time cost of the encryption process mainly includes: generation of carrier image feature codes, generation of chaotic sequences, generation of Gaussian measurement matrices, compression measurement of plaintext images, and scrambling operations in spatial and frequency domains. The time cost of decryption process mainly includes: generation of ciphertext image feature codes, generation of chaotic sequences, generation of Gaussian measurement matrices, reconstruction of planar images, and scrambling operations in spatial and frequency domains. During the decryption process, image reconstruction in compressive sensing takes up most of the time. The compression, encryption times and decryption, reconstruction times for different images are all listed in Table 14 separately. From Table 14, we can watch that firstly, for the total compression, encryption and decryption times are very little, but in decryption process, the reconstruction process costs around 99% of the total time; secondly, when the image sizes vary from 256 × 256 to 1024 × 1024, the encryption time is from 0.4191 s to 0.8916 s, but the decryption time is from 5.8835 s to 95.6098 s. Thus, the size of the image is larger, the time complexity is very higher. In the following work, we plan to adopt compressed sensing based on semi tensor product to shorten the reconstruction time.

### Analysis of chosen-plaintext attack

The chosen-Plaintext Attack and the chosen- ciphertext attack are two effective and widely adopted security attack methods in cryptanalysis^39,40^. The former assumes that the attacker has the opportunity to temporarily gain access to the encryption machine, so he/she can choose some special plaintext and obtain the corresponding ciphertext; The latter assumes that the attacker has the opportunity to temporarily gain access to the decryption machine, so he/she can choose some special ciphertext and obtain the corresponding plaintext; Then, equivalent intermediate keys can be derived from these plaintext-ciphertext pairs. Many successful cryptographic analysis cases have adopted the chosen plaintext (ciphertext) attack method.

Obviously, attackers can easily extract the intermediate ciphertext image from the ciphertext image, but they cannot crack the key streams and measurement matrices by using the method of chosen-plaintext attacks. This is mainly because the generations of the key streams and measurement matrices are related to the feature codes of the carrier image, and different carrier images have different feature codes, resulting in different generated key streams and measurement matrices. So the algorithm can resist the chosen -plaintext attack.

## Conclusion

In this article, a four dimensional discrete hyperchaotic system based on Marotto's theorem is constructed and a visual secure image encryption algorithm is proposed. The algorithm consists of two stages: In the first stage, the plaintext image is compressed and encrypted into an intermediate ciphertext image; In the second stage, the intermediate ciphertext image is embeded into the high-frequency part of the integer wavelet transform domain of the carrier image, and then performs the inverse transformation of the integer wavelet transform to obtain the corresponding visual security ciphertext image. The proposed algorithm has high security due to the fact that both the generation of the key stream and the generation of the measurement matrix are related to the feature codes of the carrier image and the fact that a multi key dynamic selection mechanism is adopted in the compression encryption stage. The experimental results and security analysis indicate that the encryption scheme has a large key space, high key sensitivity, similar histogram distribution between the carrier image and the ciphertext image.

This algorithm can resist minor noise pollution attacks, but cannot resist more severe noise pollution attacks and pruning attacks, mainly because if the noise intensity is high, the extracted feature codes from the ciphertext image may not be consistent with the feature codes from the original carrier image, resulting in poor decryption performance. Extracting carrier image feature codes as part of the encryption key can achieve one-time pad encryption effect and overcome the difficulty of key management, which is an innovative point of the paper. Next, we will continue to explore how to extract better feature codes to resist attacks from high-intensity noise pollution.

### Ethical approval and informed consent

The author declares that the images used in this article are copyrightless test images that have been authorized for publication in academic research publications. These images have been widely used in a large number of previously published academic papers without the informed consent of the image owner.

## Data Availability

The datasets used and/or analysed during the current study available from the corresponding author on reasonable request. All data generated or analysed during this study are included in this published article.
